# Piano history, aural skills, and working memory predict melodic dictation performance

**DOI:** 10.3389/fpsyg.2025.1579714

**Published:** 2025-07-29

**Authors:** Bryan E. Nichols, D. Gregory Springer

**Affiliations:** ^1^Pitch Lab, School of Music, The Pennsylvania State University (PSU), University Park, PA, United States; ^2^Center for Music Research, Florida State University, Tallahassee, FL, United States

**Keywords:** melodic dictation, interval identification, attention, auditory imagery, working memory

## Introduction

Musicians depend on the ability to classify pitches and rhythm sequences in what they hear in their own or others' playing (Halpern and Bower, 1982). *Melodic dictation*, the ability to monitor single or multi-part music and transcribe what is heard, is an important skill directly or indirectly taught to music majors as part of undergraduate study (Buonviri, 2015; Paney, 2016). Music students are subject to “ear training” for pitch and rhythm identification, dictation, and production either by individual courses or work as part of other comprehensive musicianship coursework, including error detection in conducting coursework (Silvey, 2011; Silvey et al., 2020). During melodic dictation tasks, it seems not to matter whether individuals write the transcription while listening or afterward (Pembrook, 1986), though individual strategies of melodic dictation performance have been documented including directing one's attention and prioritizing tasks (e.g., Buonviri, 2014). In the current study, we sought to understand how factors such as memory and attention, plus background variables, may also have a role in this important skill.

Students' focus of attention during melodic dictation tasks can affect their success. For example, undergraduate music majors in one study completed dictation tasks under three conditions—one in which they were directed to attend to rhythm first, one in which they were directed to attend to pitch first, and an undirected control condition (Beckett, 1997). Participants demonstrated more rhythmic accuracy on the tasks when they were instructed to attend to rhythm first. However, their pitch accuracy did not vary by condition. Paney (2016) found that directing students' attention to certain musical aspects through spoken instructions had negative effects on melodic dictation success. In that study, a control group outperformed an experimental group that received spoken instructions intended to focus their attention on specific elements. Williams (2022) reported that listeners were more successful detecting errors in three-part polyphonic music when the error was in the line on which they were instructed to focus, as compared to peripheral lines. Therefore, focus of attention seems to influence—positively or negatively—listeners' accuracy during melodic dictation and related tasks.

Musical training and other background variables have been explored in relation to melodic dictation accuracy, with some mixed findings. Surprisingly, dictation accuracy was not affected by the number of years of prior music theory study and piano skill in one study (Beckett, 1997). In contrast, years of private piano lessons served as a significant positive predictor of melodic dictation scores among collegiate music majors (Nichols and Springer, 2022). Similarly, years of experience on a secondary instrument was a significant predictor of error detection skill (Nichols and Barrett, 2024). Finally, Stambaugh (2016) reported that grades received in aural skills class—but not music theory class—were associated with higher performance on a measure of error detection. Given that various types of musical training and experience are likely to covary with melodic dictation skills, it is important for researchers to explore those relationships in studies of melodic dictation.

Cognitive factors associated with melodic dictation skill may include working memory capacity, phrase chunking, transcription ability, and the agency for selective attention (Cornelius and Brown, 2020). Related to this is the construct of mental imagery, which is the ability to imagine or picture ideas—whether visual or otherwise. For auditory imagery, the ability to imagine sounds when prompted may be important for musicians, which may be related to musical training (Gates, 2021; Halpern, 2015). Musical training seems relevant for working memory capacity, in which some types of musicians may show greater capacity than others. For example, jazz musicians outperformed classical musicians on a measure of working memory (Nichols et al., 2018), yet the role of auditory imagery in melodic dictation tasks remains unclear. Regarding the role of working memory, both verbal working memory and tonal working memory have been shown to be related to error detection performance in college musicians (Nichols and Barrett, 2024). This finding supports the potential implication of working memory in melodic dictation (Cornelius and Brown, 2020) if in fact the performance of error detection and melodic dictation performance are correlated (Larson, 1977).

Attentional flexibility is the cognitive ability to switch between tasks or levels of focus, and there is evidence that musical training enhances this ability, both in children and adults (e.g., Bain, 1978). A direct comparison showed college musicians to be superior to non-musicians (Bugos and Mostafa, 2011), but there is also evidence that degree of training is not associated with a broader measure called cognitive flexibility (Gade and Schlemmer, 2021). There is evidence of a positive correlation between cognitive flexibility and sight reading ability (Herrero and Carriedo, 2022), and sight reading has also been shown to have an association with melodic dictation (Pembrook, 1986). Further, conductors have been shown to have superior skills than pianists on some, but not all, attentional flexibility tasks (Wöllner and Halpern, 2016). Therefore, musicians may rely on attentional flexibility to perform complex tasks such as transcribing both pitch and rhythms in a melodic dictation task.

Ding et al. (2018) suggested that increased numbers of notes, but not the duration of notes, taxes working memory for musicians and non-musicians. Musicians outperformed non-musicians in a test of verbal working memory but not in a test of tonal working memory in Ding et al.'s study, suggesting different mechanisms for tonal working memory than other types of working memory. Thus, measures of memory and attention in any study of musicians might incorporate multiple modalities.

The purpose of this study was to explore the role of memory, attention, and musical background in melodic dictation. Two research questions guided the inquiry:

1. To what extent are measures of memory and attention related to melodic dictation performance? Our hypothesis was that these would be significantly related based on prior studies in focus of attention and in error detection.
2. How are prior musical experience and musical training related to melodic dictation performance? Our hypothesis was that background variables would be related to melodic dictation performance, and also that age or coursework completed would be related.

## Methods

### Participants

We sampled from undergraduate music majors at a college of music in the southeastern United States that required four semesters of written music theory and four semesters of aural skills coursework for all music degrees. Although we initially collected data from 36 participants, two participants who were enrolled for longer than the typical degree program by two standard deviations were excluded from analysis for a resulting sample size of *N* = 34. Demographic data indicated participants' age (*M* = 20.15 years, *SD* = 2.48), gender (female, *n* = 16; male, *n* = 15; non-binary, *n* = 3), year in school (freshman, *n* = 10; sophomore, *n* = 9; junior, *n* = 9; senior, *n* = 6), and primary instrument family (voice, *n* = 12; woodwind, *n* = 9; brass, *n* = 9; strings, *n* = 3; piano, *n* = 1). Participants reported completing an average of 5.09 years of study on their primary instrument (*SD* = 2.72) as well as other musical experiences, such as years of classical lessons (*M* = 5.16, *SD* = 3.25), years of jazz lessons (*M* = 0.41, *SD* = 1.26), years of experience performing in a classical ensemble (*M* = 8.47, *SD* = 2.63), years of experience performing in a jazz ensemble (*M* = 1.50, *SD* = 1.78), experience with private piano lessons (no, *n* = 23; yes, *n* = 11, *M* = 4.92 years, *SD* = 3.34), and current semester in aural skills course sequence (Aural Skills I, *n* = 3; Aural Skills II, *n* = 10; Aural Skills III, *n* = 2; Aural Skills IV, *n* = 9; completed the aural skills course sequence, *n* = 10). The onset age of musical training ranged from 9 to 30 (*M* = 14.99, *SD* = 3.85) for our sample, and the onset age of piano study ranged from 7 to 19 (*M* = 14.60, *SD* = 3.86) for those who reported experience with piano lessons.

### Procedures

We collected data from each participant individually in a research laboratory. A researcher or research assistant first administered a background questionnaire, which documented and confirmed status as a music major and continued with demographic questions related to participants' year in school and history of lessons and ensemble performance. After completing the demographic questionnaire, we screened the participants for absolute pitch. Measures of absolute pitch are common in research related to pitch perception or production abilities. Given that absolute pitch ability has been shown to be related to melodic dictation (Dooley and Deutsch, 2010), we administered a brief test of absolute pitch ability, which included 10 tones using a pre-recorded sine tones ranging from F#3 to E5 (cf. Schlemmer et al., 2005). Between each of the 10 items was a brief pause followed by dissonant clusters of notes performed with a piano timbre followed again by a brief pause before the next item. Participants continued in the subsequent data collection regardless of their performance on this test. Four participants' scores suggested absolute pitch ability. However, none of those participants' melodic dictation scores were outliers (all within 2 SD of the mean within the 95% rejection region), and none were the top scoring participants on melodic dictation. Therefore, we chose to retain these participants in our analyses, consistent with Nichols and Barrett (2024).

Following the absolute pitch screening, participants completed a battery of tests related to attention and working memory, as well as a melodic dictation task. Each of these measures are described in the paragraphs that follow in the order they were completed by participants. Data collection took ~45 min per participant, and participants took one rest break between tasks near the midpoint of the procedures.

### Tasks

#### Measures of memory and attention

##### Verbal working memory

Participants completed two measures of working memory capacity, including a common measure of verbal working memory referred to as the forward digit span test (per Talamini et al., 2016). In this test, participants hear numbers at a rate of one digit per second and are asked to repeat the numbers verbally in the order they were presented. Participants advance in duration—increasing from sets of two numbers to sets of three numbers, and if they are successful, increasing to sets of four numbers, and so on. The participant's score is the number corresponding to the highest set of numbers they recite accurately, and the participant stops the test when they do not accurately recite a series of numbers within the set. Our participants' digit span scores ranged from 4 to 9 [*M* = 5.44, *SD* = 1.19, 95% CI^1^ (5.09, 5.82)].

##### Tonal working memory

The second working memory task was a test of tonal working memory in which participants hear pairs of pitch sequences of increasing length (Nichols and Barrett, 2024), modeled after the forward digit span task in which participants' working memory is tested with increasing series of digits to be remembered (Talamini et al., 2016). Pitch sequences were presented as eighth notes at 75 bpm, and in the second sequence of each pair, half the items were presented identically, and half were presented with one different pitch, minimizing large pitch leaps or non-chord tones. After hearing each pair, participants indicated whether the pitch sequences were same or different. As in the forward digit span test for verbal working memory, the tonal working memory test progressed in terms of stimuli length. We used a presentation of 10 items of six monophonic pitches in sequence, and the order of items was randomized for each participant for this and subsequent levels of the test. Then we gave participants 10 items of eight pitches, then 10 items of 10 pitches, concluding after 10 items of 12 pitches. Unlike the forward digit span test, all participants progressed through all four levels of the test until the end. As used in Nichols and Barrett's (2024) study, the participants were scored by how many items (out of 40) they answered correctly. Among our sample, tonal working memory scores ranged from 21 to 33 [*M* = 28.24, *SD* = 3.28, 95% CI (27.15, 29.21)].

##### Auditory imagery

We chose to include auditory imagery among our measures of memory and attention based on prior studies (Gates, 2021; Halpern, 2015). Auditory imagery is conceived of here as “hearing” a sound in one's head that is not physically present, and for this construct, we used the Bucknell Auditory Imagery Scale (BAIS; Halpern, 2015). The BAIS is composed of two self-reporting subscales for Vividness (BAIS-V) and for Control (BAIS-C). Vividness is meant to describe one's ability to create an auditory image of the music (or other aural stimuli), and control is meant to describe one's ability to change the auditory image that has been created. For example, on the BAIS-V, a sample item would be “For the first item, consider the beginning of the song ‘Happy Birthday.' The sound of a trumpet beginning the piece. _____,” and participants would rate the vividness of their auditory image using a 7-point scale anchored by 1 (*no image present at all*), 4 (*fairly vivid*), and 7 (*as vivid as the actual sound*). On the BAIS-C, participants are presented an initial auditory image and are asked to change that auditory image in some way (e.g., “For the first pair, consider attending a choir rehearsal. a. The sound of an all-children's choir singing the first verse of a song. b. An all-adults' choir now sings the second verse of the song. ______”). Participants rate how easy it is to change from the initial auditory image to the second auditory image using a 7-point scale with anchors of 1 (*no image present at all*), 4 (*could change the image, but with effort*), and 7 (*extremely easy to change the image*). Both subscales (BAIS-V and BAIS-C) are composed of 14 items, and descriptive statistics for participants' BAIS-V and BAIS-C scores are provided in Table 1. Similar to Halpern (2015), we found acceptable levels of internal consistency reliability both for the BAIS-V (α = 0.79) and BAIS-C (α = 0.80), and all item-total correlations were also positive, providing further evidence of internal consistency.

**Table 1:** Descriptive statistics.

**Measure**	***M***	***SD***	**95% CI**	**Min**	**Max**
Working memory (verbal)	5.44	1.19	[5.09, 5.82]	4.00	9.00
Working memory (tonal)	28.24	3.28	[27.15, 29.21]	21.00	33.00
Attentional flexibility	66.58	24.18	[59.21, 74.57]	37.65	159.75
Auditory imagery (vividness)	5.11	0.77	[4.86, 5.35]	3.43	6.86
Auditory imagery (control)	5.50	0.77	[5.23, 5.76]	4.07	7.00
Melodic dictation (pitch)	14.65	12.35	[10.67, 18.47]	1.00	31.00
Melodic dictation (rhythm)	21.71	9.34	[18.44, 24.82]	2.00	32.00
Melodic dictation (total)	36.35	19.35	[29.70, 42.27]	6.00	63.00

*This table includes bootstrapped 95% confidence intervals using 1,000 bootstrap samples*.

##### Attentional flexibility

In addition to these two measures of working memory and the two subscales of auditory imagery, we incorporated a standard test of attentional flexibility. Defined as a form of mental flexibility, attentional flexibility involves the processes of attending to and switching between streams of information and can be measured with a simple pencil-and-paper trail making test (Reitan and Wolfson, 1985). In this test, participants are asked to connect alternating letters and numbers in ascending order, thus monitoring both “streams” at the same time (A, 1, B, 2, C, 3, etc.). Previous usage in music studies has suggested musical training is associated with faster processing speeds (Bugos and Mostafa, 2011). Our interest was not in processing speed *per se*, but whether the degree to which attentional flexibility—not only memory capacity or auditory imagery—supports the processes of melodic dictation. Durations on the trail making test ranged from 37.65 s to 159.75 s [*M* = 66.58, *SD* = 24.18, 95% CI (59.21, 74.57)].

#### Melodic dictation task

**Figure 1:**
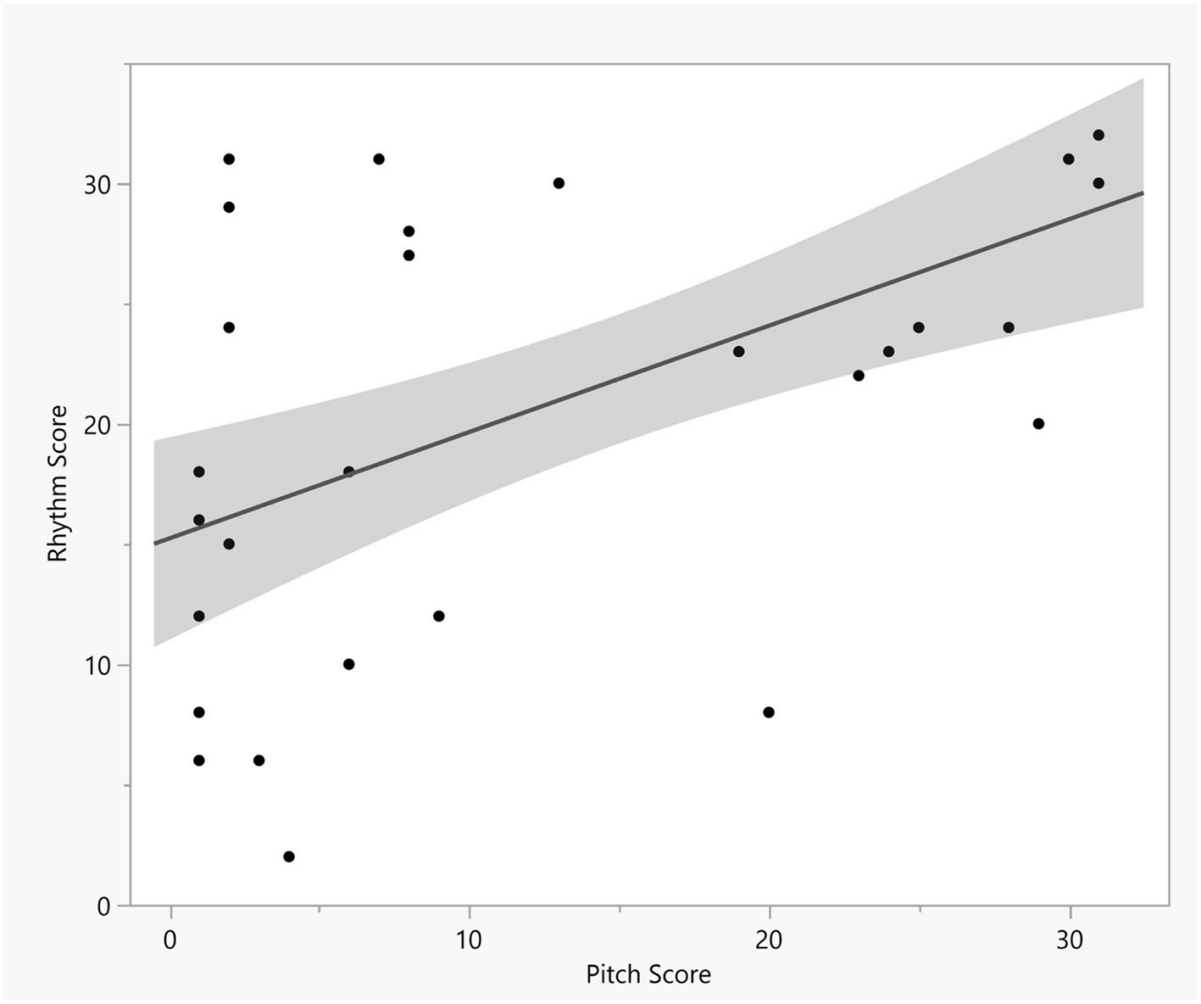
Scatterplot of melodic dictation pitch and rhythm scores. The shaded region shows the 95% confidence interval.

We included a melodic dictation task as the primary dependent measure for this study that was used in prior research (Paney, 2016). For this task, participants listened to an excerpt from the second movement (*Andante grazioso*, measures 1 through 8) of Mozart's *Divertimento No. 9 in B-Flat Major*, K. 240 performed by St. Luke's Chamber Ensemble (Mozart, 1991). We chose this task because participant scores in Paney's study were well-distributed with high scorers, moderate scorers, and low scorers. Additionally, it was in a Western diatonic tonal context, which would be familiar to participants and would be reflective of the types of melodies they would experience with dictation tasks in aural skills courses. Consistent with Paney (2016), participants heard the excerpt four times, with 30 s of silence inserted between each hearing, and participants could notate the melody at any time, including while the melody was playing. Participants notated the melody as played by the Oboe I part on a blank staff that was pre-labeled with a treble clef, key signature, time signature, and starting pitch. Paney (2016) calculated a pitch score plus rhythm score for the dictation task, and we followed the same procedure. Consistent with Paney (2016), no partial credit scores were given to strings of incorrect pitches that reflected the correct contour. The subsequent analyses are based on the total summed melodic dictation scores for each participant (pitch score + rhythm score), and the correlation of the pitch scores [*M* = 14.65, *SD* = 12.35, 95% CI (10.67, 18.47)] and rhythm scores [*M* = 21.71, *SD* = 9.34, 95% CI (18.44, 24.82)] were positive and statistically significant (*r* = 0.59, *p* < 0.001), as displayed in Figure 1.

## Results

Descriptive statistics for all measures are provided in Table 1. The following analyses are in regard Research Question 1: To what extent are measures of memory and attention related to melodic dictation performance? Attentional flexibility, as measured by the trail making test, was not significantly related to performance on melodic dictation, *r* = −0.283, *p* > 0.05 (The correlation is negative because for the trail making test, lower scores are better). Next, auditory imagery was not significantly related to performance on melodic dictation as measured by the BAIS-V (vividness, *r* = 0.141, *p* > 0.05) or BAIS-C (control, *r* = −0.005, *p* > 0.05). Finally, measures of working memory were variably related to performance on melodic dictation: performance on verbal working memory (the forward digit span test) was not significantly related (*r* = 0.243, *p* > 0.05), while performance on the tonal working memory test was significantly related to performance on melodic dictation (*r* = 0.392, *p* < 0.05). Our hypothesis was that measures of both attention and memory would be significantly related to melodic dictation based on prior studies, but this hypothesis was only confirmed for tonal working memory in the current study and not for measures of attentional flexibility or auditory imagery.

The following analyses address Research Question 2: How are prior musical experience and musical training related to melodic dictation performance? Before conducting data analyses, we screened the data to determine whether they met the assumptions for multiple regression analyses. For sample size determination, we observed the recommended 5:1 (Hair et al., 2014) and 7:1 (Miksza and Elpus, 2018) ratio of participants to predictor variables, with 28 as our minimum sample size target. Given the relatively small size of our sample (*N* = 34), we provide bootstrapped confidence intervals (Table 1) because they help improve statistical power and Type II error risk with smaller samples (LaFlair et al., 2015), thus improving generalizability. A visual inspection of scatterplots and partial regression plots suggested linear relationships. The data met the assumption of homoscedasticity as evidenced by a plot of standardized residuals against standardized predicted values, and variance inflation factors were < 10, indicating no multicollinearity (Meyers et al., 2017). We identified no univariate outliers that were more than two standard deviations from the mean, and there were no significant Mahalanobis distances, indicating no multivariate outliers. Finally, an examination of histograms and normal probability plots indicated normally distributed errors.

We conducted a two-stage hierarchical regression analysis to answer our second research question. Tonal working memory was entered into the first model, and two experience variables (years of private piano lessons and semesters of aural skills coursework) were added to the second model. Participants' melodic dictation scores served as the dependent variable. This approach allowed us to examine the unique influence of tonal working memory on melodic dictation accuracy (Model 1), while also exploring the additional influence of the experience variables in the second model. Correlations among the variables are provided in Table 2. Results indicated that the first model with one predictor (tonal working memory) was a significant predictor of melodic dictation accuracy, *F*_(1, 32)_ = 5.797, *p* = 0.022, *R*^2^ = 0.153, adj. *R*^2^ = 0.127. The second model, which also included years of private piano lessons and semesters of aural skills coursework completed, was a significant predictor of melodic dictation accuracy as well, *F*_(3, 30)_ = 5.743, *p* = 0.003, *R*^2^ = 0.365, adj. *R*^2^ = 0.301. The second model offered notably more predictive power than the previous model (Δ*R*^2^ = 0.211, *p* = 0.013). Regression coefficients and structure coefficients are displayed in Table 3. As shown in that table, all three variables were significant positive predictors of melodic dictation: tonal working memory (β = 0.406, *p* = 0.011), years of piano lessons (β = 0.410, *p* = 0.011), and semesters of aural skills coursework (β = 0.353, *p* = 0.029). Our hypothesis was that prior instrument experience in terms of lesson history or years of ensemble participation would be related to melodic dictation performance. These analyses indicated that piano history, but not other musical experience, could be used to predict melodic dictation performance.

**Table 2:** Correlation matrix.

**Variables**	**1. MD**	**2. TWM**	**3. Piano**	**4. Aural Skills**
1. Melodic dictation	–	–	–	–
2. Tonal working memory	0.392^*^	–	–	–
3. Private piano lessons	0.370^*^	0.145	–	–
4. Aural skills coursework	0.153	−0.210	−0.280	–

*Participants reported the number of years of private piano lessons completed and the number of semesters of aural skills courses completed. MD refers to melodic dictation, and TWM refers to tonal working memory*.

^*^p<*0.05*.

**Table 3:** Hierarchical regression predicting melodic dictation score.

**Model**	**Variable**	***b***	***SE***	**β**	***t***	***p***	***r***	***r_*st*_***
1	(Constant)	−28.963	27.305		−1.061	0.297		
	Tonal working memory^*^	2.313	0.961	0.392	2.408	0.022	0.392	0.649
2	(Constant)	−51.591	27.272		−1.892	0.068		
	Tonal working memory^*^	2.400	0.883	0.406	2.718	0.011	0.392	0.649
	Private piano lessons^*^	2.673	0.993	0.410	2.693	0.011	0.370	0.613
	Aural skills courses^*^	4.830	2.107	0.353	2.292	0.029	0.153	0.253

*The table includes both unstandardized (b) and standardized (*β*) beta coefficients. Structure coefficients (r*_*st*_*) are calculated as the correlation between each predictor and the outcome variable, divided by the multiple correlation coefficient (Ziglari*, *2017**)*. ^*^p<*0.05*.

## Discussion

The purpose of this study was to explore the role of memory, attention, and musical experience as in college musicians' melodic dictation accuracy. Importantly, tonal working memory was significantly related to these participants' ability to classify and transcribe a classical music excerpt, and the hypothesis was confirmed that working memory capacity in terms of the construct of tonal memory was related to melodic dictation. Our results differed from Nichols and Barrett (2024), who found that both tonal and verbal working memory were related to error detection skill, a skill that has been previously correlated to melodic dictation (Larson, 1977). Secondly, our attentional flexibility task, the trail making test, was not significantly related to melodic dictation performance. We predicted musicians may rely on attentional flexibility to perform melodic dictations based on prior evidence that expert conductors have been shown to perform higher than expert pianists on certain attention tasks (Wöllner and Halpern, 2016) and that attention to a particular musical line increases error detection in that part (e.g., Williams, 2022). Possibly expert conductors must monitor more streams of information than expert pianists as in the previous study (Wöllner and Halpern, 2016), but the monitoring of a melody line may represent a single stream rather than two components of melody information: pitches and rhythms. Because melodic dictation is a complex process that relies on some degree of bottom-up and top-down processing, we speculate that perhaps listeners integrate pitch and rhythm information in their processing of melodies during dictation task. Regardless, these data do not support the role of attentional flexibility in monitoring the two elements of pitch and rhythm in a single line melodic dictation task.

Secondly, auditory imagery was not associated with performance on melodic dictation in the current study. We used two scales for auditory imagery, one for vividness (BAIS-V) of recall of an auditory image and one for the ability to control or change the auditory imagery recalled (BAIS-C). It is logical that musicians would have a mental representation of what is heard or what they will play. However, the BAIS has been shown to have only a modest relationship to musical training (Halpern, 2015), and musicians' auditory imagery may operate quite differently from auditory imagery in the general population or on non-musical tasks. Possibly these representations are dynamic and susceptible to interruption or distractions, and there may be a limited role of metacognition for these skills which musicians exercise daily—that is, musicians may not self-report auditory imagery in yet predictable ways.

Our approach in the hierarchical regression analysis was to control for the role of working memory then to explore the experience variables accounting for significant variance in melodic dictation scores. Years of lessons on participants' primary instrument was not among these, as in previous research (Cornelius and Brown, 2020), suggesting the duration of study on one instrument may not be representative of musicians' experience globally. Rather, specific experience such as piano lesson history (a common yet variable characteristic among music majors, regardless of primary instrument) in the present study and in a previous study (Nichols and Springer, 2022) has been found to influence melodic dictation accuracy. Piano study may represent an “additive” musical experience, which is reinforced by previous findings that indicated that experience on a second instrument accounted for experience in a model of error detection skill (Nichols and Barrett, 2024). While jazz experience has been shown to reveal differences from classical experience in working memory (Nichols et al., 2018), jazz history has yet to be shown to be salient in transcribing to written music such as in melodic dictation.

The present model shows piano history and aural skills coursework to be contributing features to melodic dictation ability, which matches a previous finding for years of piano lessons (Nichols and Springer, 2022) yet differs from previous evidence melodic dictation is not related to years of piano lessons (Cornelius and Brown, 2020). Each of these variables may serve to differentiate the participants in unique ways: nearly all college musicians would be expected to have some private lessons and a rich history of ensemble experiences during their undergraduate experiences. Beyond that, they may differ in terms of musical history in that some may have started an instrument such as piano at a very young age whereas others may have initiated formal music study later. Then, experience in aural skills coursework contributes to practice in classifying what is heard or played. This supports a previous assertion that students' grades from aural skills class was associated with higher error detection performance (Stambaugh, 2016), a skill that has been correlated to melodic dictation (Larson, 1977). However, we acknowledge the low correlation between aural skills class level and melodic dictation score. Thus, we interpret the significant effect for the aural skills variable in our regression: these results can be interpreted cautiously as the relationship between aural skills coursework and musicianship skills such as melodic dictation has not been widely explored.

## Limitations

Results of this study are subject to certain limitations. These results are based on a sample of 34 collegiate musicians, and although this sample size exceeded our target, it is relatively small. We provide bootstrapped confidence intervals (Table 1) because bootstrapped resampling approaches help to improve statistical power and reduce the Type II error risk. However, we acknowledge that the generalizability of findings would be improved with increased sample size (Hair et al., 2014). Attentional flexibility may not have played a significant role in melodic dictation in the current study based on the monophonic transcription such as in our test (cf. Paney, 2016). However, a task that required participants to monitor more than one melodic line or more than one part at a time may rely more on attentional flexibility than the current paradigm required—an area for future research. Attentional flexibility must be measured carefully in future research, and we acknowledge that conflation with processing speed may be a concern for testing this ability. Next, we asked these participants to perform a pitch and rhythm dictation task in which some people may have attended more to rhythm thereby reducing their pitch score in an unrepresentative way of their pitch dictation ability, and vice versa. Event density should be considered an important factor in melodic dictation and musical tasks generally. Cornelius and Brown (2020) reported a statistical interaction between the number of repetitions and the number of leaps in the melody, claiming both of these would affect the working memory capacity in dictation tasks and presumably, error detection and other related tasks (Larson, 1977). Thus, these results may be particularly dependent on the stimuli we chose for this study (Paney, 2016). Further to this, Ding et al. (2018) found the number of notes but not duration of notes to affect working memory performance for musicians and non-musicians; this also offers implications for the type and frequency of notes in excerpts selected for use in this study.

## Future research

Additional exploration of the role of attentional flexibility as it relates to monitoring pitches and rhythms is warranted. Further, selective vs. divided attention as a salient construct for all musician tasks including error detection, melodic dictation, and performing music has the potential to inform the pedagogy of performance and the development of musical skills at all age levels. We use the previous findings from Wöllner and Halpern (2016) to promote the possibility that musicians may rely on attentional flexibility to perform complex tasks such as transcribing both pitch and rhythms in a melodic dictation test in some ways. How exactly participants chose to attend to pitch and rhythm elements was not documented as part of this study, and we believe there is potential for learning about how musicians manage these two elements simultaneously may yield tools for young musicians to efficiently and accurately demonstrate greater proficiency. Additionally, Dooley and Deutsch (2010) asserted that absolute pitch is highly related to melodic dictation, yet the participants in the present study indicating the likelihood of possessing absolute pitch did not reveal this tendency which should be explored in future research. Importantly, the influential role of piano study on melodic dictation performance as observed in this study raises the question of *what kind* of piano study would result in improved dictation skills (e.g., classical solo repertoire, functional chordal accompaniment, jazz improvisation, etc.). In future investigations, researchers might gather data on the types of piano study in which participants have engaged to further understand what kinds of piano study are most advantageous to melodic dictation success. Additionally, the starting age of musical training—which may be associated with piano study at an earlier age than other instruemtns—may be useful to document in future studies.

The presence of musical training—as existed in our sample of college of musicians—has been associated with faster processing speeds (Bugos and Mostafa, 2011). Our interest was not in processing speed *per se*, but whether the degree to which mental processing in terms of attentional flexibility—not only memory capacity or auditory imagery—influences the processes of melodic dictation. There is evidence that the focus of attention determines where in the score a musician will be more successful at processing pitch information (Williams, 2022). Given that musicians must monitor multiple elements of music (pitch, rhythm, expression, and sometimes diction) as well as multiple sensory inputs (auditory feedback from ensemble, visual feedback from conductor, haptic feedback from one's own instrument, etc.), attentional flexibility is an important area for continued inquiry in music education research.

## Implications for music education

Success in melodic dictation is elusive and may vary individually and as students vary in background/training and play different instruments. Possibly, melodic dictation should be taught after assessing pitch vs. rhythm skills to design an individual plan for instruction (i.e., all students are not the same). Because aural skills coursework seems to influence performance, instructors might offer individual plans in conjunction with student-directed goals and tracking from year to year and as it relates to fewer notation-based styles. Previous research suggests jazz musicians differ from classical musicians in working memory capacity, and possibly teaching and performing in a range of styles can benefit global musician skills more by providing both visual and aural modes of presentation and responding. Students with piano experience on classical solo repertoire may have quite different experience than those using functional chordal accompaniment or jazz improvisation, and these features can all be explored in piano lessons and classes, and in future research.

Given that tonal working memory was a positive predictor of melodic dictation accuracy, those who teach courses that involve dictation and related aural skills tasks may consider designing learning experiences that will further develop students' tonal working memory. For example, following the protocol from previous working memory studies, students could practice identifying short sequences of pitches that gradually increase in number as they are successful (e.g., starting with sequences of four pitches, then five, then six, etc.). Melodic dictation seems to be a developmental skill, based on the findings of the current study. Therefore, these types of learning experiences will allow students multiple opportunities to practice transcribing sets of pitches that gradually increase in number and complexity.

The pitch and rhythm sub-scores for melodic dictation were strongly correlated suggesting that overall, participants who do well on one element will do well on the other. That seems important because it suggests musicians may not attend to just one of the two. Or, alternatively, it could suggest the absence of a strategy for approaching the task. Regardless, common tests of melodic dictation sum the two scores, as did ours. However, melodic dictation tasks of increasing complexity in terms of number of parts at a time may require strategies such as choosing one element (e.g., pitch) over another in order to be successful. We maintain the evidence supports the conclusion that more and more types of musical experience may all contribute to musicianship, including piano lessons, secondary lesson experience (Nichols and Barrett, 2024), jazz history (Nichols et al., 2018), and other coursework or informal music experience.

## Conclusion

We put forth these results as contributing evidence for the limited role of working memory capacity in musicians' skills; musicians rely on the ability to monitor and maintain pitches, rhythms, and other elements in their working memory. Working memory capacity alone does not account for all the variance in musicians' melodic dictation scores. The data support the assertion that musicians rely on a range of experience, training, practice, and coursework to support their musical productivity above and beyond the working memory capacity that musicians draw upon. Melodic dictation of classical excerpts is but one indicator of the ability to classify and document what is heard. Thus, it offers only a limited view of an increasingly broad view of musicianship, and individual students might become more invested in aural-only modes of transmission as well as visual-aural modes such as is typically presented in classical styles. However, dictation does provide one indicator of students' pitch and rhythm processing, which is helpful for instructors at diagnosing learning difficulties and assessing students' aural skills.

## Data Availability

The raw data supporting the conclusions of this article will be made available by the authors, without undue reservation.

## Footnotes

^1^All 95% confidence intervals reported in the paper are bootstrapped using 1,000 bootstrap samples.
